# Family Planning for Strangers: An Experiment on the Validity of Reported Contraceptive Use

**DOI:** 10.1371/journal.pone.0136972

**Published:** 2015-08-31

**Authors:** Guy Stecklov, Alexander A. Weinreb, Mariano Sana

**Affiliations:** 1 Department of Sociology and Anthropology, The Hebrew University of Jerusalem, Jerusalem, Israel; 2 Department of Sociology and Population Research Center, University of Texas at Austin, Austin, Texas, United States of America; 3 Department of Sociology, Vanderbilt University, Nashville, Tennessee, United States of America; Tulane University School of Public Health, UNITED STATES

## Abstract

Sterilization levels reported in the Dominican Republic appear well above what we would normally expect given prevailing patterns in the region. We suspect that the use of strangers as interviewers—the normative approach in data collection in both developed and developing country settings—may be partly responsible for this result, and may underlie a long history of bias in family planning data. We present findings from a field experiment conducted in a Dominican town in 2010, where interviewer assignment was randomized by level of preexisting level of familiarity between interviewer and respondent. In our data, sterilization use is higher when the interviewer is an outsider, as opposed to someone known to the respondent or from the same community. In addition, high sterilization use is correlated with a propensity of respondents to present themselves in a positive light to interviewers. These results call into question the routine use of strangers and outsiders as interviewers in demographic and health surveys.

## Introduction

The Dominican Republic (DR) has the highest levels of female sterilization in Latin America. Across six waves of Demographic and Health Survey (DHS) data collected between 1986 and 2007, sterilization among women aged 15–49 increased from 22 percent to 35 percent, before falling slightly in 2013. Given that the prevalence of non-permanent (modern) methods increased from 8 to 26 percent of women over the same period, this means that since the mid-1980s, female sterilization has been the dominant method of choice for some two-thirds of current users of family planning.

The central question that we address in this paper is whether these estimates of sterilization in the Dominican Republic (DR), and possibly contraceptive prevalence in the DR more generally, are exaggerated. At first glance, this may sound like an odd question. No red flags about the accuracy of data on sterilization or contraceptive use in the DR have been raised by respected demographers or public health researchers who have used these data [1–3]. On the contrary, on standard indicators, DR DHS data quality looks good. They have high response rates typical of DHS surveys [4], a smooth and steady increase in prevalence of both sterilization and non-permanent methods (and associated fertility decline), and the literature finds a relatively high concurrence (>60%) between husband’s and wives’ reported contraceptive use [1]. In more qualitative terms, too, notwithstanding the heavy influence of the Catholic Church in the DR [5], and conservative attitudes towards virginity and premarital sex among women [6], researchers have documented the longstanding availability of female sterilization [7]. They have also largely commended the DR authorities since the 1960s for creating a receptive context for family planning programs [8,9], even if the relative absence of method mix available to women—particularly the over-reliance on female sterilization—has been seen to dampen overall contraceptive use [6,10,11].

Dig a little deeper into demographic theory, data, and data collection practices, however, and we find sufficient grounds for asking whether sterilization is over-reported. First, while low levels of non-response, high response reliability, and relatively high husband-wife concurrence on reported contraceptive use capture certain dimensions of data quality, they shed little light on the validity of the reported contraceptive use, that is, how accurately actual contraceptive behavior is measured. Indeed, since DHS tends to employ the same data collection methods across multiple waves, it is quite conceivable that high reliability is hiding systematically high levels of response bias. Imprecise measurement is a widely noted and much lamented problem with reproductive health data in non-Western settings, and attempts to redress it have underlain many innovations in data collection methodology [12–16].

Second, it has long been recognized that in populations with low contraceptive prevalence, social desirability or conformity biases can propel women to underreport contraceptive use [12,17,18]. It is now equally clear that in populations with moderate levels of contraceptive prevalence, women can demonstrate their identity as modern, virtuous, and “aspirational” in a number of ways [19], including by admitting use of contraception [20]. By extension, it is reasonable to suggest that in populations with high contraceptive prevalence, social desirability bias may lead some to over-report contraceptive use, especially when the interviewers come from, or are thought to represent, the most cosmopolitan, modern and advanced part of the country. In the DR, in particular, since female sterilization has long been perceived as the go-to contraceptive method, it is reasonable to suggest that either social desirability or conformity bias may lead non-users (or users of other methods) to claim use of female sterilization. Few want to be seen as non-users where nonuse signals cultural backwardness.

**Fig 1:**
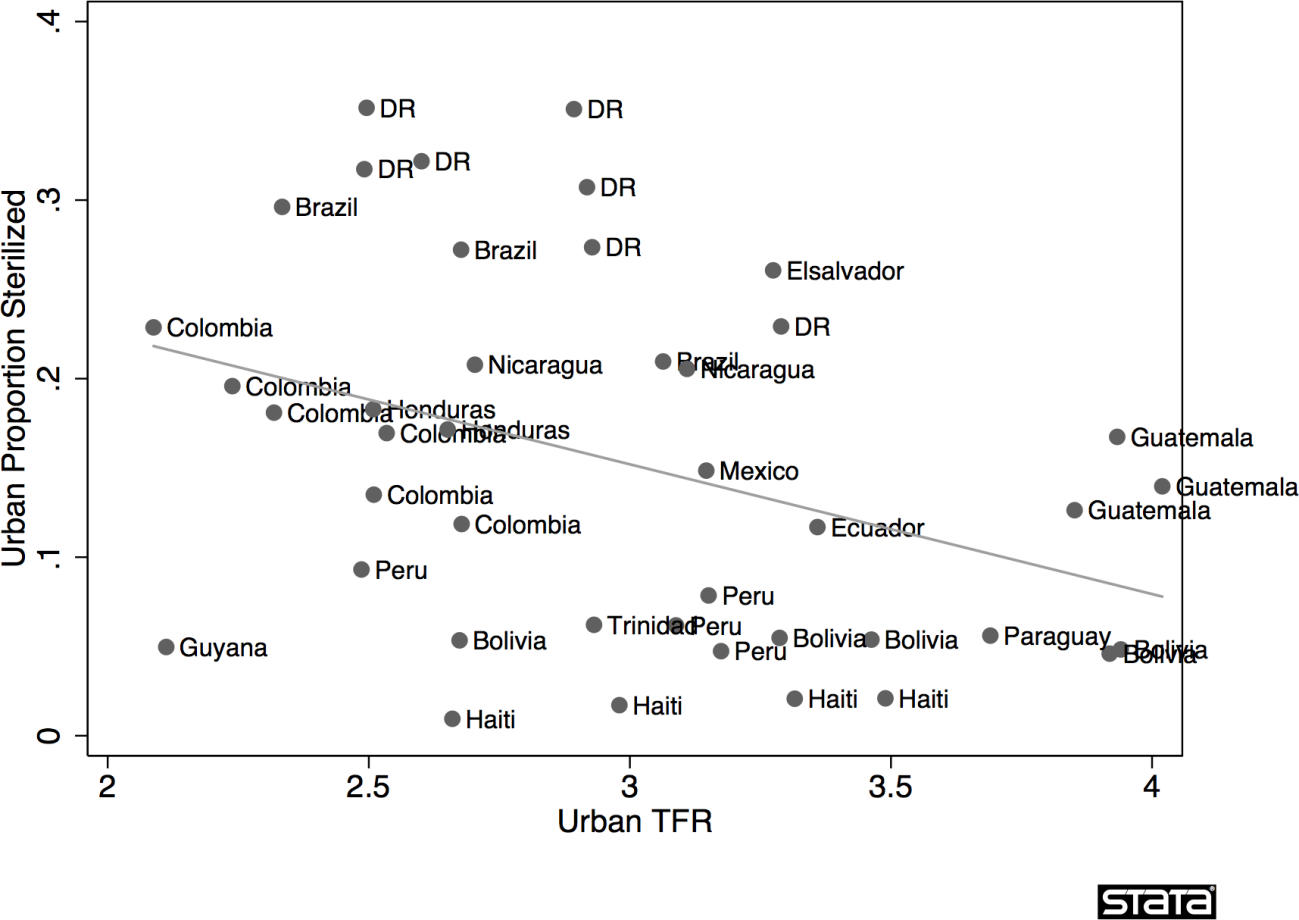
Scatterplot for Reported Sterilization and TFR in Urban Latin American DHS data, 42 surveys from 15 countries, including OLS Regression Prediction Line (R^2^ = 0.14; p = 0.015).

Finally, augmenting each of these arguments is an important empirical point. When the DR is compared to other urban areas in Latin American countries in terms of the relationship between TFR and reported sterilization—notably Brazil, Nicaragua and Colombia, which also have high levels of female sterilization—the DR looks like an outlier. We show this in Fig 1, where we plot multiple waves of DHS survey data to show the fraction of urban women reporting sterilization relative to the TFR for the DR (7 waves) and for equivalent DHS data from Bolivia (5 waves), Brazil (3), Colombia (6), Ecuador (1), Guatemala (3), Guyana (1), Haiti (4), Honduras (2), Mexico (1), Nicaragua (2), Paraguay (1), Peru (4), and Trinidad (1). With an urban TFR ranging from roughly 2.5 to 3.3 across the seven waves, the DR’s fertility is higher than that of many countries in our sample. Yet the percentage of women reporting sterilization in the DR—the most effective contraceptive method—is easily the highest. Its maximum is five percentage points greater than the next highest rate (Brazil, with lower fertility), and far higher than most other countries with similar or lower fertility levels.

This unusual juxtaposition of high sterilization and relatively high TFR could be explained by complex differences across a range of proximate determinants. However, any or all of these proximate determinants may themselves be subject to misreporting. We offer an alternative explanation for the DR’s outlier status on the sterilization/fertility nexus. We argue that the exceptional levels of reported sterilization use are in part a product of misreporting. We show that misreporting on sterilization in the DR appears to be driven by a particular type of interviewer effect, specifically, the level of interviewer-respondent familiarity. As we detail below, data collection protocols associated with the DHS, World Bank and other large international survey organizations, prescribe the use of “stranger” interviewers, that is, interviewers who have no preexisting social relationship with the respondents. Typically, these interviewers are also “outsiders,” that is, not from the neighborhood or survey site. In the DR, we argue that sterilization reported to these outsiders is likely exaggerated.

We base our argument on the results of a methodological experiment that we implemented in a single urban setting in the DR. The design included a fully randomized and interpenetrating sample of respondents and interviewers with different (and known) levels of interpersonal familiarity that predated the survey interaction. Consistent with our expectations, we find that lower and more realistic levels of female sterilization are collected by local interviewers, both those who have a prior relationship to their respondents and those who do not, than by outsiders, the standard type of interviewer employed by the DHS and surveys in general.

### Using insiders, outsiders, and local strangers to conduct surveys

We outline our argument for why the use of “strangers” as interviewers may be a poor choice in many societies–particularly non-Western ones—elsewhere [21,22]. Here our focus is on explaining the DR’s outlier status on the sterilization/fertility nexus. We argue that even if "(s)urveys often venture into areas that people do not ordinarily discuss with strangers" [23], and strangers receive "the most surprising openness" [24], this in itself tells us nothing about the validity of the information that is received. On *theoretical* grounds alone, there are good reasons for supposing that in more socially dense and less socially diverse settings—more typical of less developed societies—respondents will give stranger-interviewers less valid and/or reliable information than they would to local interviewers they already know. There are less established traditions of trusting strangers in these types of settings [25–27], in part because fewer non-threatening strangers (doctors, lawyers, therapists, teachers, social workers, and so on) are around to pose questions. This is a structural characteristic of developing societies. Relative to their more developed counterparts, these are not “interview societies” [28]. There is also some nascent *empirical* evidence of this effect in non-experimental studies focused on the relative benefits of insider- and stranger-interviewers [21, 22], local- and outsider-interviewers [25, 26], and where the empirical focus is on the data quality effects of other types of social connectedness [27]. Though there is variability in the magnitude of these effects across different types of questions, the general finding is that familiarity between interviewers and respondents sometimes augments data quality, and it never reduces data quality. This is contrary to standard data collection norms in survey research, although the use of “resident enumerators” is now being implemented in a number of surveys in Africa as part of the Performance Monitoring and Accountability 2020 Project.[29]

## Design of the experiment and methods of analysis

The DR, the focus of our analysis, is a middle-income country with 9 million inhabitants, a GNI per capita of 7,150 USD, life expectancy at birth of 68 years, and 64 percent urban. More important for our research, fertility rates are estimated to have begun falling in the mid-1960s from a TFR of over 7 children per woman to near replacement levels in a few decades [30], making it a compelling setting for research on how women report during an interview on their contraceptives practices.

We use data from a methodological survey fielded in 2010 in a medium-sized town in the DR that we refer to here as San Benito. Confidentiality commitments require that we not name the town, but our selection criteria focused on finding a small to medium sized town that was not completely enveloped by a larger city but was affected by social and economic modernization. We hoped this way to capture a setting that combined a degree of anonymity co-existing with small-town familiarity.

We emphasize four key elements of the research design. First, all interviewers and respondents were women. Second, the survey instrument includes questions on reproductive behavior, including contraceptive use and abortions, that we use to study how responses to questions on reproductive behavior are affected by the extent of pre-existing levels of interviewer-respondent familiarity. Third, we distinguish three types of interviewers according to levels of interviewer-respondent familiarity—insiders, local-strangers, and outsiders. Fourth, we randomly assign these three types of interviewers to respondents.

Broadly speaking, the three types of interviewers break down by familiarity with place and familiarity with person. “Outsiders” represent the classical type of survey interviewer. They are both non-local and have no prior relationship with the respondent or the community. "Insiders" lie at the other end of the continuum. They are both local and familiar with the respondent and the community. "Local-strangers" capture an intermediate status: interviewers who are from the same town or community (e.g., a particular neighborhood within a city) but have no personal relationship with the respondent or their family. Although there is less emphasis on this intermediate category in classical theory, it is an increasingly visible type of interviewer selection strategy, in particular in projects adopting a Community-based Participatory Research (CBPR) approach. More important here, it helps us identify which of the axes of difference appears to matter more for data quality—familiarity with place or person—and whether this varies by the substantive focus of the question.

Our analytic focus in this study is on reported contraceptive use, in general, and on female sterilization, the dominant method, in particular. In the course of the survey, where a respondent reported current use of a method to avoid pregnancy, she was asked what method she was currently using. The response options that we defined as modern method use included female sterilization, male sterilization, the pill, IUD, injections, implant/norplant, male condom, female condom, and emergency contraception. From this list of responses, we derived a three-category variable representing non-use of any modern method, use of any non-permanent modern method, and female (along with two cases of male) sterilization.

Since the study design aimed to examine the effect of levels of interviewer-respondent familiarity that preceded the interview, formal sampling was preceded by the selection of interviewers. Female interviewer positions were advertised in the San Benito area and, after a written test and informal interview that assessed the interactional skills of each applicant, 32 were selected for training. In addition, eight female professional interviewers were brought in from Santo Domingo, the capital city. All these trainees—both the locals and outsiders—were trained in the same room for a period of five days. At the end of the training, in order to make sure that interviewers from both groups were sufficiently well trained and to reduce heterogeneity across the local and outsider interviewer groups, the trainees were given a test to evaluate their understanding of the survey instrument and interviewing protocols. In all, six of the 32 locals and two of the eight outsider interviewers were not used in the fieldwork. This process both helped maintain standards and also increased confidence that levels of ability were not radically different across the two groups going into the field.

As a first step in designing a fully randomized and interpenetrating sample of respondents and interviewers with different (and known) levels of interpersonal familiarity, each of the local interviewers was asked to list the names and approximate addresses of all women (in the eligible age group) whom they knew well or very well. No incentive was promised to women based on the number of names provided. Over a period of five days, all local interviewers were taken around town by a member of the research team in order to find and mark each household they claimed to know on detailed census maps acquired from the DR Central Bureau of Statistics (1:900 scale). These maps list every building in the town and immediate surroundings, with special markings for number of stories, mixed-use business and residential dwellings, and buildings under construction. The maps were only three-years old and highly accurate in general. Where missing houses were found, they were hand-drawn onto the hard copy of the maps (irrespective of whether or not they belonged to someone on the interviewers’ lists).

We refer to the complete set of houses containing an eligible respondent that at least one of the local team of interviewers knew (n = 641), as the “claimed” stratum of the sample, while the rest of houses on the map (n = 4,834) formed the “unclaimed” stratum. Every one of the claimed households was randomly assigned to one of the three types of interviewers—insiders, local-strangers, and outsiders. Where a household assigned to an insider had been claimed by more than one local interviewer, the interviewer was randomly selected from among those claimants. Subsequently, we drew a random sample of unclaimed households (n = 1,161) and randomly assigned them to each of our 30 interviewers. This latter group formed the “unclaimed” stratum in our sample. By design, it is not possible to have insider interviews in the unclaimed stratum.

The interview protocol, approved by the Vanderbilt University Institutional Review Board (IRB) in 2010, was carefully designed to promote the randomized assignment of eligible women respondents across the three types of interviewers at the household level. Due to the minimal risks posed to participants, the IRB permitted us to work with verbal consent to reduce the possibility of altering the nature of pre-existing trust between respondents and interviewers—a key element of our experiment. Following the first stage of the interview, during which informed consent was obtained, a roster of women of eligible age (20–50) in the household was created. Where there was more than one eligible woman per household, interviewers used a simple turn-taking algorithm to randomly select one of them. In all cases, if the selected woman was not available, interviewers returned up to three times to attempt to conduct the interview.

Two minor types of contamination arise from this randomization at the household level. First, in five cases, a household member was claimed by one of our local interviewers and this household was subsequently selected in the random assignment to be interviewed by the interviewer who made the claim. However, the interviewer noted at the end of the interview that she did not know the particular respondent at all. An alternative strategy focusing on individuals—while interesting in and of itself—was beyond the scope of this first experiment.

A second form of contamination involved 10 cases where a local-stranger was sent to interview a household but she turned out to be very familiar with the respondent. In each of these situations, additional measures of interviewer-respondent familiarity collected during the interview were used to check whether these two forms of contamination have any effect on estimates. They do not. There is high degree of overlap between the initial assignment variable and actual reported familiarity in the survey, and use of either measure generates the same substantive results [31]. Finally, our main findings build primarily on the distinction between local and outsider interviewers–a distinction unaffected by either form of contamination.

Two additional steps were taken to assure a proper identification of insider/stranger/outsider response effects rather than confounding these effects with some other spatial or temporal factor. First, interviewers operated in all areas of town (they moved between supervisors, each of whom was assigned responsibility for a different section of town). Second, local interviewers alternated between insider and outsider roles–depending on whether they were interviewing one of their own claimed households, a household claimed by someone else, or a household in the unclaimed stratum. This means that local interviewers did not, for example, conduct all their insider-interviews first, and only then move on to other households–or vice-versa. Administering the survey in that order would potentially confound insider-related response effects with an interviewer’s changing skill levels or fatigue.

Table 1 presents detailed descriptive data about the intended sample and detailed outcomes, completed interviews as well as the household response rate in each of the two strata. Overall, 1207 interviews were conducted, with 536 completed in the claimed stratum and 671 in the unclaimed stratum. Overall, significantly higher completion rates were achieved in the claimed than unclaimed stratum – 92.4 and 72.3 percent, respectively (p<0.05)–an unsurprising difference given that claimed households were more likely to be inhabited and contain an eligible respondent. Some five percent–mostly pregnant women—did not provide information on family planning and were removed from later analyses (n = 65)–a percent similar across interviewer types. More relevant to the central research question of this paper, interview completion rates were broadly similar across the categories of interviewers: within the claimed sample locals showed slightly higher but insignificant (p = 0.22) rates whereas within the unclaimed sample they were higher–though insignificantly (p = 0.67)—for outsiders.

**Table 1:** Household response rates and count distribution of outcomes, by strata and type of interviewer. Dominican Republic Interviewer-Respondent Familiarity Project Data, 2010.

	**Claimed Stratum**			
	**Local-Insider**	**Local-Stranger**	**Outsider**	**Subtotal**
**Household Response Rate** ^*^	0.932	0.932	0.902	0.924
** ** **Count of Outcomes**				
**A. Completed**	219	179	138	536
**B. No Eligible Member Present**	10	8	8	26
**C. Household Absent**	5	3	0	8
**D. Refused**	6	5	7	18
**E. Dwelling Vacant/Not Dwelling**	1	2	2	5
**F. Other**	19	13	16	48
**Total**	260	210	171	641
	**Unclaimed Stratum**			
	**Local**		**Outsider**	**Subtotal**
**Household Response Rate** ^*^	0.719		0.732	0.723
** ** **Count of Outcomes**				
**A. Completed**	455		216	671
**B. No Eligible Member Present**	160		77	237
**C. Household Absent**	23		5	28
**D. Refused**	18		2	20
**E. Dwelling Vacant/Not Dwelling**	101		50	151
**F. Other**	33		21	54
**Total**	790		371	1,161

* Household Response Rate = (A / (A+B+D))

Our analyses build on the randomized design and use OLS, logistic and multinomial logistic regression models. We begin with null models without additional control variables, because the covariates themselves might alter our findings if they are also influenced by interviewer familiarity. We subsequently introduce controls typically associated with reproductive behavior to help to reduce potential noise in the data, although our main findings all stand alone without these controls, and the controls are purposefully omitted from models presented in the last two tables. The controls include age and education of respondents, number of children reported by the respondent and a wealth index based on the first principal component taken from a list of durable goods reported by the respondent [32,33]. Successive models are used to test robustness of our findings to different sample subgroups. In particular, we later evaluate whether differences in reporting contraceptive use by level of interviewer-respondent familiarity are similar among women who we objectively measured to have a higher tendency to lie on an unrelated variable (note that the terms “lies” or “liar” are chosen for the sake of brevity of text without any pejorative intent). Finally, we control in all our models for role-restricted interviewer effects in our survey by correcting for clustering of respondents within interviewers. This correction is typically effective, particularly with more than 30 groups as in our case [34], at inflating the standard errors of estimators to compensate for intra-cluster correlated errors within interviewers in our data (rho = 0.65).

## Main experimental results

A traditional way to evaluate the strength of the experimental design is to compare descriptive statistics across the interviewer categories as shown in Table 2. Although differences in these descriptive characteristics might be due to design flaws or to systematic biases that may be present across interviewer categories, the summary statistics provide some further confidence in the strength of our experimental design. The sample size is here restricted to 1,127 cases, given that a small number of cases were missing on one or more of the explanatory variables in our analyses and more importantly because 65 women who reported being pregnant (and not using contraception) were excluded from the analysis—this did not vary across type of interviewer. The data show very small differences in values for age, education, children ever born and wealth across the respondent types, a small fraction of the size of the standard deviations. We also provide the results of tests of equality in the last column for whether the type of interviewer predicts differences in the covariates, with controls for the clustering of respondents within individual interviewers. These tests are estimated using linear regression (F-Test) for the continuous covariates and logistic regression (χ^2^) for the four binary outcomes. The results show that the null hypothesis of no relationship between type of interviewer and the covariates cannot be rejected for age, education, children ever born, and union status. However, the difference between interviewers in the value of reported wealth is significant, if barely, and indicates that insider interviewers found higher levels of wealth than both local-strangers and outsiders. It is worth noting that this contrast is no longer significant (p = 0.224) once insiders and local-strangers are collapsed and are compared as a single category (“locals”) with outsiders, nor does it remain significant (p = 0.874) once we control for whether the observation originates from our claimed or unclaimed sample–a control included in all our subsequent regression models given the fundamental difference in how the samples were drawn. Finally, the most provocative finding that emerges in Table 2 is the very meaningful and significant differences in the reported use of family planning across the interviewer categories, with outsiders finding much higher levels of use than found by the two types of local interviewers. Overall, the randomization appears successful in randomly allocating most attributes–at least those observables we include in our analysis as control variables–into the three separate groups of interviewers and it is successful for all covariates once we collapse our interviewers into two groups. At the same time, the descriptive results also indicate that reported use of family planning differs strongly across interviewer types. It is to this issue that we now turn.

**Table 2:** Respondent descriptive statistics and statistical tests for differences across interview categories. Dominican Republic Interviewer-Respondent Familiarity Project Data, 2010 (n = 1,127).

	Type of Interview			
	Local-Insider	Local-Stranger	Outsider	P-values for testing of equality of means or proportions
	Mean/(SD)	Mean/(SD)	Mean/(SD)	
**Age**	35.359	34.705	34.639	0.731
	(8.522)	(8.719)	(8.980)	
**Education** ^**a**^	0.660	0.596	0.594	0.418
	(0.475)	(0.491)	(0.492)	
**Children Ever Born**	2.150	2.377	2.310	0.244
	(1.479)	(1.529)	(1.540)	
**Wealth Index**	0.198	-0.024	-0.064	0.048
	(0.960)	(0.999)	(1.033)	
**In Union**	0.655	0.701	0.639	0.151
	(0.476)	(0.458)	(0.481)	
**Modern Method FP Use**	0.432	0.527	0.642	0.002
	(0.497)	(0.500)	(0.480)	
**Count**	206	586	335	

^a^ Education refers to at least secondary education versus less than secondary (reference)

### The Role of Familiarity

Our main analysis begins by evaluating the impact of local-stranger and outsider interviewers, relative to insiders (the reference category), on reported family planning use. We then re-estimate our models in a simpler specification where insiders and local-strangers are combined into a single, joint “local” category. We only show empty models for our initial specifications, where local-insiders and local-strangers are distinguished, but our results are equally clear once we combine them into a single *local* category. Our findings for the six alternative specifications are shown below in Table 3.

**Table 3:** Logistic Regression Estimates of Effect of Interviewer Familiarity on Reported Use of Modern Family Planning, by Strata, Dominican Republic Interviewer-Respondent Familiarity Project Data, 2010.

	Claimed Sample			Full Sample		
	Reference Category			Reference Category		
	Local-Insider		Local	Local-Insider		Local
	(1)	(2)	(3)	(4)	(5)	(6)
**Local-Stranger**	0.333	0.362		0.308	0.367	
	(0.220)	(0.240)		(0.217)	(0.230)	
**Outsider**	0.761**	1.007***	0.844**	0.794**	1.003***	0.721**
	(0.282)	(0.296)	(0.279)	(0.248)	(0.260)	(0.225)
**Age**		-0.0110	-0.0120		-0.0165*	-0.0172*
		(0.0117)	(0.0118)		(0.00791)	(0.00801)
**Education** ^**a**^		0.0723	0.0657		-0.0430	-0.0415
		(0.257)	(0.253)		(0.137)	(0.135)
**Wealth Index**		-0.0713	-0.0750		-0.00312	-0.00373
		(0.0893)	(0.0897)		(0.0644)	(0.0648)
**Children Ever Born**		0.477***	0.483***		0.417***	0.420***
		(0.115)	(0.114)		(0.0897)	(0.0895)
**In Union**		0.913***	0.897***		1.033***	1.024***
		(0.217)	(0.222)		(0.178)	(0.179)
**Claimed Sample**				-0.105	0.113	-0.0352
				(0.170)	(0.185)	(0.156)
**Constant**	-0.274	-1.594***	-1.397**	-0.169	-1.400**	-1.034*
** **	(0.215)	(0.483)	(0.460)	(0.271)	(0.461)	(0.403)
**N**	507	507	507	1127	1127	1127
**LL**	-345.6	-305.8	-307.1	-764.5	-685.0	-686.6
**Chi2**	7.499*	76.11	66.96	12.11**	109.5***	100.8***

Standard errors in parentheses

^^^ *p* < 0.10

* *p* < 0.05

** *p* < 0.01

*** *p* < 0.001

^a^ Education refers to completion of at least secondary education versus less than secondary (reference)

The first three columns on the left, using the claimed sample, show whether local-strangers differ from local-insiders (the reference category) in reported family planning use. While separation of insiders and local-strangers is theoretically salient, our results clearly show that this distinction is not empirically meaningful in this first analysis. Yet, in contrast to the lack of difference between local-insiders and local-strangers, a very large difference is found between local-insiders and outsiders. To put these coefficients (in the models with controls) in perspective, using the parameters from the model on the claimed sample, when all covariates are set at their mean values, the highly significant coefficient on outsider (p<0.001) indicates that while the estimated probability of reported use is 41.3% for local-insider interviewers, it is 50.3% for local-strangers (not a statistically significant difference relative to local-insiders) and 65.8% for outsider interviewers.

The lack of difference between insiders and local-strangers argues for a more parsimonious model focusing on the difference between locals and outsiders. We separately replicate the models using first the claimed sample and then the full sample, but no longer distinguishing between local-insiders and local-strangers (see Columns 3 and 6 in Table 3). This simplification provides a consistent and clear result: there are highly significant gaps in reported use of family planning between what is reported to outsider interviewers and what is reported to local interviewers. We find that the estimated probability of reported family planning use is 49.7% when interviewed by a local and 67.0% when interviewed by outsiders. This highly significant difference is consistent regardless of whether we use the claimed sample or the full sample. In fact, a separate test on the entire sample but including an interaction between the claimed sample indicator and the outsider indicator provides no support for there being any difference in the effect of the outsider variables across the claimed and the unclaimed samples (p = 0.49).

While our first analysis focuses on family planning use in general, a particularly important question in the Dominican context is whether familiarity affects reported use of sterilization. To address this question, we employ multinomial logistic regression, with no use of modern contraception as the reference category and use of non-permanent modern method and use of sterilization as alternative outcomes. Our results, with specifications similar to those in Table 3, are presented in Table 4. We first show the results on the claimed sample (columns 1–3) before showing the results on the full sample (columns 4–6).

**Table 4:** Multinomial Logistic Estimates of Effect of Interviewer Familiarity on Reported Use of Modern Non-Permanent Methods or Sterilization versus Non Use, by Strata, Dominican Republic Interviewer-Respondent Familiarity Project Data, 2010.

	Claimed Sample			Full Sample		
	Reference Category			Reference Category		
	Local-Insider		Local	Local-Insider		Local
	(1)	(2)	(3)	(4)	(5)	(6)
	**Non-Permanent Methods**					
**Local-Stranger**	0.167	0.105		0.262	0.163	
	(0.305)	(0.306)		(0.294)	(0.299)	
**Outsider**	0.701^	0.576	0.531	0.609^	0.569^	0.448*
	(0.417)	(0.386)	(0.373)	(0.335)	(0.298)	(0.193)
**Age**		-0.145***	-0.146***		-0.142***	-0.143***
		(0.0229)	(0.0232)		(0.0158)	(0.0158)
**Education** ^**a**^		0.161	0.158		0.0396	0.0400
		(0.354)	(0.352)		(0.222)	(0.221)
**Wealth Index**		-0.137	-0.136		-0.0347	-0.0334
		(0.135)	(0.136)		(0.0993)	(0.100)
**Children Ever Born**		0.0439	0.0471		0.106	0.108
		(0.131)	(0.132)		(0.108)	(0.108)
**In Union**		1.802***	1.799***		1.609***	1.604***
		(0.317)	(0.318)		(0.291)	(0.291)
**Claimed Sample**				-0.0470	0.188	0.116
				(0.222)	(0.222)	(0.184)
**Constant**	-1.266***	1.888**	1.959**	-1.219***	1.766**	1.937***
	(0.219)	(0.690)	(0.691)	(0.308)	(0.603)	(0.522)
	**Sterilization**					
**Local-Stranger**	0.420	0.467		0.336	0.460^	
	(0.271)	(0.310)		(0.250)	(0.260)	
**Outsider**	0.794**	1.364***	1.148**	0.887**	1.270***	0.908**
	(0.285)	(0.400)	(0.365)	(0.292)	(0.362)	(0.322)
**Age**		0.0810***	0.0796***		0.0518***	0.0510***
		(0.0183)	(0.0179)		(0.0110)	(0.0110)
**Education** ^**a**^		0.196	0.178		-0.0227	-0.0219
		(0.297)	(0.289)		(0.153)	(0.151)
**Wealth Index**		-0.0752	-0.0907		0.0130	0.00787
		(0.109)	(0.110)		(0.0709)	(0.0695)
**Children Ever Born**		0.878***	0.888***		0.659***	0.662***
		(0.189)	(0.184)		(0.116)	(0.115)
**In Union**		0.741*	0.723*		1.000***	0.986***
		(0.329)	(0.338)		(0.229)	(0.231)
**Claimed Sample**				-0.133	0.0809	-0.0979
				(0.173)	(0.198)	(0.176)
**Constant**	-0.737**	-6.735***	-6.468***	-0.604*	-5.147***	-4.687***
** **	(0.240)	(0.946)	(0.889)	(0.303)	(0.660)	(0.583)
**N**	507	507	507	1127	1127	1127
**LL**	-511.5	-371.1	-372.5	-1152.0	-894.2	-895.9
**Chi2**	8.315^	153.6***	145.7***	13.74*	677.6***	434.0***

Standard errors in parentheses

^^^ *p* < 0.10

* *p* < 0.05

** *p* < 0.01

*** *p* < 0.001

^a^ Education refers to at least secondary education versus less than secondary (reference)

The top panel of Table 4 compares use of non-permanent methods to non-use. We find that there are no statistically significant differences by type of interviewer in the claimed sample, but that the increased sample size in the full sample combined with similar effect sizes seen in the claimed sample, show significant findings. In this case, turning to the full sample, we still find no difference between local-strangers and local-insiders. However, the differences between outsiders and locals emerge as significant and substantial.

In the bottom panel of Table 4, we compare reported sterilization to non-use. The results are similar to, but stronger than, those in in the top panel. We find no significant differences between insiders and local-strangers, which allows both categories to be combined (Columns 3 and 6), although the difference is marginally significant in the full sample. Thus, while there are consistent effects between the two types of local interviewers, our results do not offer any conclusive evidence as to their impacts. On the other hand, when we use the combined local category, we find strong and significant differences in responses on sterilization when interviewers are outsiders versus when they are locals. The main lessons to emerge from Table 4 are that 1) local-insiders are mostly indistinguishable from local-strangers and the two categories can be aggregated for our purposes; 2) interviews with outsiders produce much higher levels of reported use of non-permanent methods and sterilization when compared to data collected by locals; and 3) The effects of outsiders on reported use of non-permanent methods and sterilization are similar across the claimed and unclaimed samples.

### Testing for honesty

Our results so far highlight a major gap between contraceptive use reported to locals and outsiders and this difference is broadly consistent for non-permanent methods and sterilization. Given the random assignment, we can be confident that the difference in reported use has to do with the effect of familiarity rather than unobservable factors. But, these results on their own do not tell us whether the gap is due to respondents over-reporting to outsiders or under-reporting to locals. In particular, given the primacy of sterilization in terms of its presumed role in the DR contraceptive mix, we focus on determining which reported sterilization levels appear more valid: those reported to locals or those reported to outsider interviewers.

Our survey instrument was specifically designed to shed light on self-presentational mechanisms that might be driving differences in reported behaviors. We turn to two sets of questions aimed at gauging respondent’s self-presentation that were asked about three-quarters through the interview. One short set focused on personality traits and the second on knowledge. The first set enabled respondents to present themselves as an *ideal* personality–someone that either “never criticized anyone” or “never felt sad.” We created an indicator for whether a respondent claimed yes on either one of these two questions. Needless to say, while some people may in truth *never* criticize or feel sad, the assignment of interviewers is randomized so the measured difference in response is expected to be dependent on interview type.

In the second, knowledge-based question set, the interviewer listed, one at a time, the names of nine people, introduced as “famous” personalities, asking respondents whether they had heard of each of them in turn. Seven were very well-known public figures: they included Michael Jackson, Fidel Castro, the most famous Dominican sportsman, and the First Lady of the DR. Two were fictitious personalities enabling us to test “over-claiming” [35]. This question set therefore evaluates one’s self-presentation in terms of general knowledge. Where a respondent claims to have recognized a fictitious person we refer to them as having told a “soft-lie,” which we interpret as indicative of a person’s susceptibility to social desirability bias. We also included a measure of the “real people” that respondents reported to recognize, providing a useful further control. As noted above, in the Dominican context, we expect social desirability to be associated with higher levels of reported contraceptive use.

Our next analysis assesses the effect of interviewer-respondent familiarity on both measures of self-presentation: the likelihood of reporting an ideal personality and of having told a soft lie. We then estimate a third model on soft lies, controlling for personality self-presentation and the count of real people that respondents claim to have known. Our results, seen in Table 5, show that in reporting their personality, respondents do not self-present any differently to outsider or local interviewers (first column- note that one additional case is dropped in this analysis due to nonresponse on the question of known personalities). In contrast, turning to the knowledge-based test of soft lies, we find a large difference by familiarity in whether people reported knowing fictitious persons listed by the interviewer (second column). The difference indicates that respondents are much more likely to produce a soft lie on these questions when interviewed by outsiders. In substantive terms, this difference in lying is very large: following a simple transformation of the logistic regression coefficients into probabilities we see that the estimated probability of at least one lie is 22% when interviewed by a local and 41% when interviewed by an outsider. Lies in this case offer evidence that in interactions with outsider interviewers, respondents are far more likely to present themselves in a way that makes them appear smarter and more worldly. The fact that this effect is robust to efforts to control for both ideal personality and real people noted in the questionnaire strengthens our argument that soft lies capture distinct dimensions of bias produced by the type of interviewer.

**Table 5:** Logistic Regression Test of Effect of Outsider versus Local Interviewers on Knowledge and Self-Presentation. Dominican Republic Interviewer-Respondent Familiarity Project Data, 2010.

	Ideal Personality	Soft Lies	Soft Lies w/ Controls
**Outsider** ^**c**^	-0.387	0.890***	0.846***
	(0.276)	(0.216)	(0.231)
**Ideal Personality**			0.267^
			(0.139)
**Real People**			0.335***
			(0.0894)
**Constant**	0.206	-1.109***	-3.433***
	(0.131)	(0.123)	(0.658)
***N***	1126	1126	1126
**LL**	-775.3	-645.1	-635.7
**Chi2**	4.992	17.84***	27.72***

Standard errors in parentheses

^^^ *p* < 0.10

* *p* < 0.05

** *p* < 0.01

*** *p* < 0.001

^c^ Reference category is locals.

Our results have shown that respondents report far more use of contraception, including sterilization, when interviewed by outsiders than by locals. Two questions arise from these findings. One is whether this effect is due to variation in interviewer-respondent familiarity—our argument—or to the greater professional experience of outsider interviewers. Because there were no professional interviewers among the insiders, it is not possible to directly distinguish interviewers’ professional experience from their level of insiderness. However, both age and occupation can shed some light on whether we are primarily identifying job experience and training. Among the local interviewers, we found that neither age nor occupational background (categorized into housewives, students, and employed) status of interviewers–our possible proxies for experience–affect sterilization reporting (p = 0.131 and p = 0.101, respectively). Similarly, among outsider interviewers, we tested for the role of age and found no effect either (p = 0.630). We conclude that differences in interviewer-respondent familiarity remain the more likely source of this variability in reporting.

Our second and more central concern is that we cannot *directly* validate respondent questionnaire answers to determine which type of interviewer receives more accurate responses. To overcome this constraint, we use two alternative approaches to *indirectly* establish validity. First, we examine whether responses given to outsiders tend to be exaggerated in ways that allow the respondents to present themselves in a better light. The findings from Table 5, including the use of the soft lie variable, provide an indirect validation test and support the interpretation that responses to outsiders are more likely to be biased.

A second test is to see whether we can use both familiarity and soft lies together to better understand higher or lower reporting of family planning use. To simplify this analysis, our three-category measure of family planning use (no use as the reference category; use of a non-permanent method; and sterilization) is regressed on a categorical variable that combines level of familiarity and whether at least one soft lie is reported. The reference category for this explanatory variable is outsider interviews where there were no reported lies on the fictitious people test. We then test for the effect of outsider interviews with reported lies about knowing fictitious people, local interviews with reported lies, and local interviews with no reported lies, on reported use of either type of family planning versus non-use of any method. As noted above, we do not include control variables both because of the randomized treatment design and because the effect of the lies themselves might be captured by over- or under-reporting on any of the covariates if those who lied about fictitious people also systematically lied on other questions as well.

Two very clear patterns emerge in the results, shown in Table 6. The first is that the largest (most negative) coefficients are on locals, indicating that regardless of whether respondents lied or not, there was much less reported use of family planning in interviews with locals, consistent with our earlier results. These effects are particularly strong and statistically significant for non-permanent methods, and for sterilization either not significant for “lie to locals” or only marginally significant in the case of “no lie to locals.” Unsurprisingly, given the coefficients and standard errors in Table 6, if we contrast “lie to locals” versus “no lie to locals” the difference is clearly significant (p<0.05).

**Table 6:** Multinomial logistic regression estimates of effect of Lies and Familiarity on Reported Modern Family Planning Use, Dominican Republic Interviewer-Respondent Familiarity Project Data, 201.

	Full Sample
***Modern Non-Permanent Methods***	
**Lie to Outsiders** ^b^	-0.0466
	(0.238)
**No Lie to Locals** ^b^	-0.428*
	(0.214)
**Lie to Locals** ^b^	-0.432^
	(0.244)
**Constant**	-0.545***
	(0.139)
***Sterilization***	
**Lie to Outsiders** ^b^	0.311*
	(0.158)
**No Lie to Locals** ^b^	-0.493^
	(0.278)
**Lie to Locals** ^b^	-0.514
	(0.366)
**Constant**	0.202
	(0.238)
***N***	1127
**LL**	-1152.2
**Chi2**	23.67**

Standard errors in parentheses

^^^ *p* < 0.10

* *p* < 0.05

** *p* < 0.01

*** *p* < 0.001

^b^ Reference category is No lie to outsiders.

In addition to the lower levels reported to locals, there is a very noticeable increase in reported use of sterilization when interviewed by outsiders who “lie.” Thus, for outsiders, lies are associated with more reporting of sterilization use but no such difference is apparent for non-permanent methods. In substantive terms, among those interviewed by outsiders, the predicted probability of reporting sterilization for those who claimed to know a fictitious person is 49.5% while for those who did not make this claim, it is 41.4%. In contrast, among those interviewed by locals, the probability is roughly 33% regardless of whether they lie or not. These gaps strengthen our claim that at least part of what explains the apparent over-reporting of sterilization among respondents in our sample—and potentially in the DR more broadly—is the increased tendency to present oneself as smarter and more worldly. However, this is only observable in interviews with outsiders. At least in terms of sterilization, our main focus, local interviewers do not trigger the same inclination to over-report.

## Western paradigms of data collection

Family planning continues to occupy a central role in the development agenda, both among groups focused on reproductive health per se and those advocating for the broader benefits of contraceptive use [36,37]. Since the early KAP surveys, one of the central problems in this work has been getting the numbers right [38]. Even with the considerable investments in high quality surveys such as the World Fertility Surveys and the Demographic and Health Surveys—the latter undoubtedly a gold standard among international survey data collection efforts and one whose core substantive focus is contraceptive use—that is not a trivial task.

In fact, mismeasurement of key parameters in sample surveys in LDCs has been long lamented. One response to this lament has been to express skepticism about the utility of these types of data and to push, with varying degrees of vigor, for their abandonment [17,39–43]. Another response has been to emphasize the role that posthoc methods such as instrumental variables procedures [44,45], latent variable models [46–48], or multilevel models [49–51] can play in “cleansing” and/or testing mismeasurement, thereby avoiding biased results. Yet another approach has focused on getting the data “right” from the start. This includes proposals to eliminate interviewers from the data collection process entirely—since these middlemen are suspected to cause a substantial part of the total survey error—as in various techniques involving self-administered questions [13,14,52,53]. It also includes incorporating methods more commonly associated with qualitative data collection, such as shifting from fully structured and standardized questionnaires towards more conversational styles of interviewing, semi-structured interviewing, immersion in the field setting, and flexibility in the design of survey tools (e.g., the "ethnosurvey" and “microdemography”) [12,54].

Our arguments are closest to this last approach. Following a rich qualitative literature on the relationship between researcher-informant familiarity and the quality of social interaction and data [55–63], we suggest that survey researchers may be able to take advantage of locals’ "entanglement in family and party interests" and of the fact that they are bound to respondents by norms of reciprocity, or at least seen to be bound to the local community. Outsider-interviewers, in contrast, have no social leverage over the respondent or vice versa. This difference can affect the quality of data collected in the interview.

A larger methodological discussion is clearly at play here. We think that in developing country settings, in particular, discussions of “role-independent” interviewer effects [64] rarely move beyond examining the effects of an interviewer’s gender on respondent’s reports. This is too restrictive. Such debates should also include a much broader set of relational parameters that lie at the core of Simmel’s idea of “entanglement.” This is the central quality he uses to distinguish a true stranger—typically an outsider—from someone rooted in a community and its underlying web of relationships. Social scientists have long asserted that the stranger-driven culture of urbanized western societies—the primary proving ground for survey methods—is not a dominant mode of cultural expression in global terms. This in itself suggests that the places where outsiders tend to collect better data than insiders may be outnumbered by the places where the reverse is the case.

In summary, getting the data right is not trivial, but it is a task that we need to take seriously. Our analysis of family planning data in the DR—using data from a randomized experiment—suggests that across a range of disciplines involved in health-related research, we may be defaulting too quickly to the wrong type of interviewer. More hopefully, our analysis also demonstrates that by acknowledging cross-cultural variability in interactional norms, and allowing it to inform our choice of interviewers, we can improve the accuracy of reporting. In this paper we have shown that this helps us generate more realistic estimates of levels of sterilization than those implied by the DR’s unusual position on the sterilization-fertility ranking. Based on these results, which parallel others we describe elsewhere [21,22,65], we think there are strong grounds for experimenting with more intimacy in interviewer-respondent familiarity than is currently the case. This is the case even with the ongoing global shift towards more dense and highly urbanized populations. Our findings demonstrate that in small or medium sized urban settings—the dominant form of urban settlement—interviewer-respondent familiarity may not necessarily harm and may even be beneficial in terms of data quality. Whether interviewer-respondent familiarity has the same effect in large cities in developing countries, or even in megacities, is an open question. Nonetheless, the bottom line is clear: so long as our goal is to collect the most accurate data, we should not restrict ourselves to a methodological monoculture in which we only employ outsider-interviewers. We need to diversify the pool from which we draw our interviewers, as we have learned to diversify other methodological choices.

## Supporting Information

S1 File STATA Limited DRIRFP Data file for replication. (DTA)

S2 File STATA Program Do-file to replicate tables. (DO)
